# Evaluation of Pulmonary Hypertension in Chronic Obstructive Pulmonary Disease

**DOI:** 10.7759/cureus.21828

**Published:** 2022-02-02

**Authors:** Gaurang M Aurangabadkar, Ajay V Lanjewar, Ulhas S Jadhav, Saood N Ali, Pankaj B Wagh

**Affiliations:** 1 Respiratory Medicine, Jawaharlal Nehru Medical College, Wardha, IND

**Keywords:** copd, chronic obstructive pulmonary disease, pulmonary hypertension, pulmonary artery systolic pressure, gold staging, echocardiography

## Abstract

Background: Pulmonary hypertension (PH) is frequently underdiagnosed and under-evaluated in chronic obstructive pulmonary diseases (COPD) patients. As PH is linked to a high rate of mortality from disease-related complications like cor pulmonale, it is critical to have a unified approach to diagnosing and treating it with the aim of improving the patient's quality of life and prognosis in terms of therapeutic considerations. Early identification of this comorbidity in patients with COPD can lead to early initiation of treatment and better prognostic implications. This study was undertaken with the aim of estimating the prevalence of PH in COPD as well as assessing any statistically significant associations between the severity of PH and the severity of COPD.

Methods: This was an observational study undertaken at the Department of Respiratory Medicine, Acharya Vinoba Bhave Rural Hospital (AVBRH) in Wardha, Maharastra, India, from August 2019 to September 2021. A total of 100 patients diagnosed with COPD on the basis of spirometry were evaluated with two-dimensional (2D) echocardiography to screen for echocardiographic signs and evidence of PH and severity staging of PH if present.

Results: In our study, out of 100 diagnosed cases of COPD, the prevalence of PH was found to be 40% (40 patients) and amongst these, mild, moderate, and severe PH was seen in 26 patients (26%), nine patients (9%), and five patients (5%), respectively. The frequency of PH in moderate COPD was 25% and in severe COPD was 51.5%.

Conclusions: PH was found in almost half the COPD patients in the study. Also, the degree and frequency of PH increased with the increase in COPD severity and this was found to be statistically significant.

## Introduction

The most common comorbidities associated with chronic obstructive pulmonary disease (COPD) are systemic hypertension, ischemic heart disease, Type 2 diabetes mellitus, respiratory infections, nutritional deficiencies, PH, cor pulmonale, pulmonary thromboembolism, depression, and anemia [1]. Cardiovascular disease is a major co-morbidity in COPD [2]. Systemic, as well as pulmonary hypertension (PH), is the principal cardiovascular complication encountered in COPD. COPD-associated PH is widely being recognized to be a contributing entity in the development of disease manifestations, morbidity, and eventual mortality [3]. The natural history of the disease is severely affected by the presence of PH which leads to frequent exacerbations, rapid decline in lung functions, persistent hypoxemia, and premature morbidity and mortality.

The frequently undetected and chronic PH eventually causes right ventricular (RV) hypertrophy, dilatation, and subsequent RV failure [4]. Pulmonary arterial pressures are of significant importance in the prognosis of COPD. In a review study of indicators of prognosis, pulmonary arterial pressures greater than 20 mm of Hg were found to significantly predict mortality at five years [5]. Furthermore, the factor of superior prognostic importance in COPD patients who were prescribed long-term oxygen supplementation was observed to be the magnitude of pulmonary artery pressures rather than the pulmonary function test (PFT) or arterial blood gas (ABG) abnormalities [6]. Thus, prompt diagnosis of PH in COPD patients and early intervention have great prognostic implications for the patient. The “Gold Standard” for the evaluation of pulmonary vascular pressures as well as for diagnosing primary PH, and evaluation of congenital heart diseases prior to cardiac or lung surgeries and heart-lung transplantation surgery is still right heart catheterization (RHC) [7]. It is not, however, feasible to perform RHC in every patient given the high prevalence of COPD, the high prevalence of PH in COPD, significantly associated risks due to the invasive nature of the procedure, and cost issues [6]. Along with its diagnostic role, two-dimensional (2D) echocardiography is also useful as a screening tool for patient groups that are considered high risk as well as for prognostic monitoring, assessing the stability of the disease, and treatment response. Patients with PH can be comprehensively evaluated for its cause and can be accurately grouped in their respective categories with the help of echocardiography. Thus, the present study was undertaken to screen COPD patients for PH with the help of Doppler echocardiography, to estimate the prevalence of PH in COPD, and to assess any statistically significant associations between the severities of PH and COPD

## Materials and methods

The study was an observational study conducted between August 2019 to September 2021 in the Department of Respiratory Medicine at Acharya Vinoba Bhave Rural Hospital, Wardha, India. Institutional Ethics Committee - Datta Meghe Institute Of Medical Sciences (Deemed University) issued approval DMIMS (D.U.)/IEC/ Sept-2019/8344. A structured case history proforma was used for recording the history, clinical signs, radiological findings, electrocardiographic and echocardiographic findings, and laboratory tests of the patients.

Inclusion and exclusion criteria

All male and female patients above 18 years of age, who were diagnosed with COPD on the basis of spirometry post-bronchodilator forced expiratory volume in one second/forced expiratory volume (FEV1/FVC) ratio less than 0.7 as per Global Initiative for Chronic Obstructive Lung Disease (GOLD) guidelines [8], and were willing to participate in the study after giving informed consent, were included in the study

Patients diagnosed with pulmonary tuberculosis (present or past), bronchial asthma, acute left ventricular failure, ischemic heart disease, cardiomyopathies, primary PH, and other comorbid conditions and patients with poor echocardiography window were excluded from the study.

Data collection

A PFT was performed using an RMS Helios 401 PC-based spirometer (Recorders & Medicare Systems P Ltd., Haryana, India) in compliance with the American Thoracic Society/European Respiratory Society (ATS/ERS) standards. The test yielded the following results: FEV1, FVC, FEV1/FVC ratio, slow vital capacity (SVC), and maximal voluntary ventilation (MVV). The main parameters utilized to stage COPD patients according to GOLD guidelines were post-bronchodilator FEV1, FVC, and the FEV1/FVC ratio. For those with FEV1/FVC ratio less than 0.7, the post-bronchodilator FEV1 values were used to stage the severity of COPD as per GOLD guidelines as mild, moderate, severe, and very severe as given in Table 1. To rule out the possibility of bronchial asthma, all patients underwent post-bronchodilator spirometry (metered-dose inhaler (MDI) salbutamol 400 mcg with spacer).

**Table 1:** Staging of COPD according to the GOLD guidelines COPD: Chronic Obstructive Pulmonary Disease; GOLD: Global Initiative for Chronic Obstructive Lung Disease Source: Global strategy for the diagnosis, management, and prevention of chronic obstructive pulmonary disease: 2021 report [8].

GOLD STAGE	COPD SEVERITY	FEV_1_ (% predicted)
GOLD 1	Mild	≥ 80% predicted
GOLD 2	Moderate	50% to 79% predicted
GOLD 3	Severe	30% to 49% predicted
GOLD 4	Very Severe	< 30% predicted

Two-Dimensional Transthoracic Doppler Echocardiography

Resting 2D transthoracic Doppler echocardiography was performed on all patients by skilled cardiologists. The machine utilized was an Affiniti 70 Ultrasound system (Phillips Healthcare, Amsterdam, Netherlands) equipped with a PureWave S5-1 Broadband sector array transducer (Phillips Healthcare, Amsterdam, Netherlands). The color flow Doppler echocardiography was able to identify and measure the tricuspid regurgitant jet velocity; the peak jet velocity was quantified using the continuous wave Doppler approach without using intravenous contrast.

In the absence of right ventricular outflow obstruction, the modified Bernoulli equation was employed to determine right ventricular systolic pressure (RYSP), which was deemed to be equal to systolic pulmonary arterial pressure (PAP).

RVSP = trans-tricuspid pressure gradient (TTPG) + right atrial pressure (RAP) = systolic PAP (mmHg)

RVSP= 4Vmax^2^ + RAP

where trans-tricuspid pressure gradient is 4Vmax^2^ (V= peak velocity of tricuspid regurgitation in m/s).

RAP was estimated to be 5, 10, or 15mm of Hg based on inspiratory variations in inferior vena cava (IVC) dimensions as follows:

RAP = 5mm of Hg (in case of complete IVC collapse)

RAP = 10mm of Hg (in case of partial IVC collapse)

RAP = l5mm of Hg (in case of no IVC collapse)

In this study, PH was defined as systolic PAP greater than 25 mmHg, according to the definition of pulmonary arterial hypertension [9]. PH was categorized as mild, moderate, or severe based on systolic PAP values of 30-50, 50-70, and >70 mmHg, respectively. The following values were selected using the Chemla formula [10]: mean pulmonary arterial pressure (mPAP) =0.61 systolic PAP + 2 mm Hg. The following systolic PAP values were generated by placing the values of mPAP for mild, moderate, and severe PH as 25-35, 35-45, and >45 mm Hg, respectively.

Other Investigations

The other investigations that the patients underwent were: sputum for acid-fast bacilli and bacterial culture, hemoglobin, total leucocyte count, differential leukocyte count, liver and kidney function tests, ECG on admission, ABG analysis on admission, chest x-ray posteroanterior view, CT Thorax (only in patients with clear indications for the same on the basis of primary chest radiological findings).

Statistical analysis

For numerical outcomes that were regularly distributed, the Mean ± Standard deviation was determined. The Mann-Whitney U test was used to determine the Mean ± SD of demographic variables among patients with and without PH. To make group comparisons, non-parametric tests (Kruskal-Wallis test) were utilized. Strength of Association was calculated by point-biserial correlation. For comparing the mean of subgroups, Fishers’s exact test and unpaired t-test were used. To compare non-numerical variables, the Chi-square test was used. IBM SPSS Statistics for Windows, Version 20.0 (Released 2011. IBM Corp., Armonk, New York) was used. The significance level was kept at P-value ≤ 0.05 level.

## Results

Characteristics of the study population

Age and Gender Distribution

Out of the total 100 patients with COPD, 70% were males while 30% were females. The age distribution of the study participants is shown in Table 2.

**Table 2:** Age distribution of the study population

Age	Frequency	Percentage	95% CI
40-49 Years	5	5.0%	1.9-11.8%
50-59 Years	17	17.0%	10.5-26.1%
60-69 Years	43	43.0%	33.3-53.3%
70-79 Years	31	31.0%	22.3-41.1%
80-89 Years	4	4.0%	1.3% - 10.5%

Occupational Distribution, Symptoms, and Clinical Signs

In our study, 79 patients (79%) were involved in agriculture-related professions while 21 patients (21%) were involved in the non-agricultural sector. 95 participants (95%) gave a history of exposure to biomass fuel smoke for household cooking and heating purposes. The summary of the symptoms is given in Table 3 and the clinical signs are summarized in Table 4:

**Table 3:** Summary of symptoms in the study population

SYMPTOMS	PRESENT	ABSENT
Cough	87 (87.0%)	13 (13.0%)
Expectoration	62 (62.0%)	38 (38.0%)
Haemoptysis	3 (3.0%)	97 (97.0%)
Breathlessness	93 (93.0%)	7 (7.0%)
Fever	30 (30.0%)	70 (70.0%)
Weakness and Fatigue	37 (37.0%)	63 (63.0%)
Chest pain	45 (45.0%)	55 (55.0%)
Weight And Appetite loss	14 (14.0%)	86 (86.0%)

**Table 4:** Summary of clinical signs in the study population

SIGNS	PRESENT	ABSENT
Pallor	36 (36.0%)	64 (64.0%)
Icterus	0 (0.0%)	100 (100.0%)
Cyanosis	7 (7.0%)	93 (93.0%)
Clubbing	10 (10.0%)	90 (90.0%)
Use of accessory muscles	29 (29.0%)	71 (71.0%)
Pedal Oedema	28 (28.0%)	72 (72.0%)
Raised JVP	6 (6.0%)	94 (94.0%)
Tachycardia	68 (68.0%)	32 (32.0%)
Tachypnea	38 (38.0%)	62 (62.0%)

Comorbidities in the study population

In our study, it was observed that 42 patients (42%) had systemic hypertension and 21 patients (21%) had diabetes mellitus, while 37 patients (37%) had no history of either. Thus, systemic hypertension was found to be the leading comorbidity (42%) followed by diabetes mellitus (21%).

Radiological findings

The radiological findings of the study participants are summarized in Table 5.

**Table 5:** Summary of radiological findings in the study participants

Radiological findings	Frequency
Normal radiological findings	24 (24.0%)
Emphysema	59 (59.0%)
Cavity	3 (3.0%)
Pleural effusion	7 (7.0%)
Pulmonary artery thrombosis (On CT Thorax)	2 (2.0%)
Pulmonary hypertension (On CT Thorax)	12 (12.0%)
Bronchiectasis	13 (13.0%)

Distribution of PH and its severity

**Figure 1:**
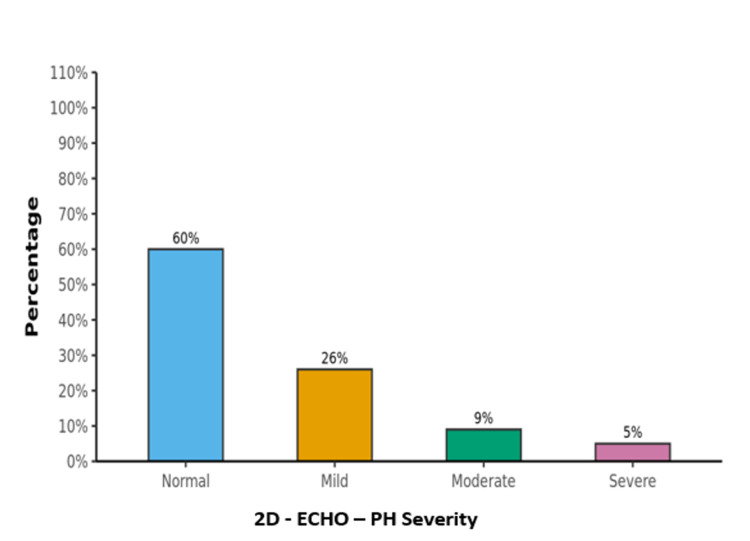
The distribution of PH severity by two-dimensional echocardiography 2D ECHO: two-dimensional echocardiography; PH: pulmonary hypertension

In our study, out of 100 patients with COPD, 40 patients were diagnosed as having PH on 2D echocardiography. Out of these 40 patients, the distribution of severity of PH was as follows: mild PH in 26 patients, moderate PH in nine patients, and severe PH in five patients. The distribution of PH and its severity by 2D echocardiography is shown in Figure 1.

Summary of 2D echocardiographic findings

The echocardiographic findings of the study participants are summarized in Table 6.

**Table 6:** Summary of echocardiographic findings in the study participants 2D ECHO: two-dimensional echocardiography

2D-ECHO PARAMETERS	FINDINGS
Right atrial dilatation	Present in 26 patients (26.0%)
Right ventricular dilatation	Present in 13 patients (13.0%)
Pulmonary artery dilatation	Present in 42 patients (42.0%)
Systolic pulmonary artery pressure (mmHg)	(Mean ± SD): 32.66 ± 17.15
Left ventricular ejection fraction (%)	(Mean ± SD): 58.60 ± 4.10
Detectable tricuspid egurgitation	Present in 39 patients (39.0%)
Pulmonary hypertension	Present in 40 patients (40.0%)

Association between GOLD staging and the severity of PH on echocardiography

**Figure 2:**
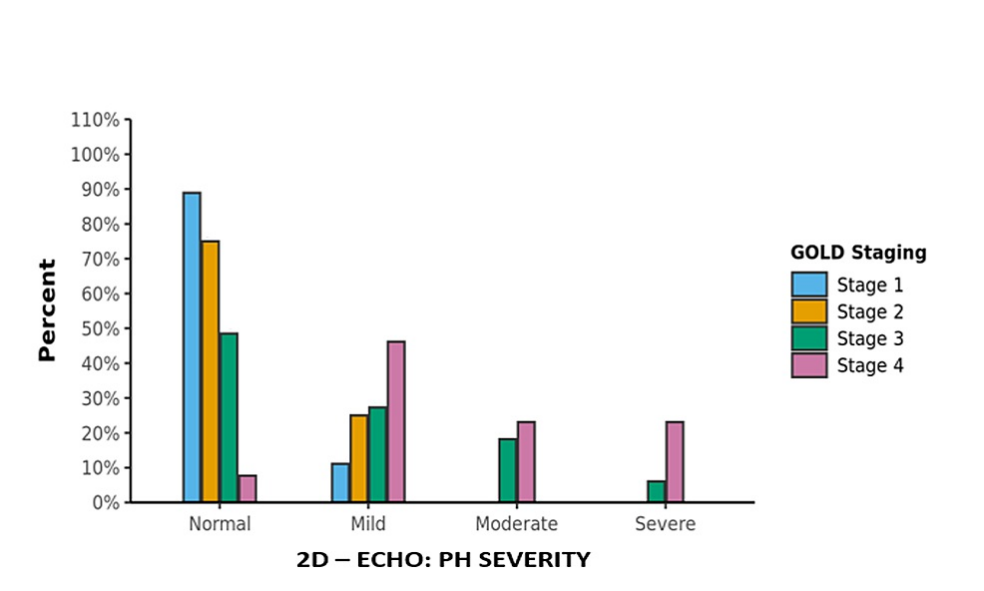
Association between GOLD staging and PH severity GOLD: Global Initiative for Chronic Obstructive Lung Disease; PH: pulmonary hypertension

There was a significant difference between the four GOLD stages in terms of distribution of severity of PH and was statistically significant (χ2 = 36.195, p = <0.001). Participants in the GOLD Stage 1 had the largest proportion of normal 2D echocardiography studies (88.9%). Participants in the GOLD Stage 4 had the largest proportion of mild to severe PH (Table 7, Figure 2).

**Table 7:** Association between GOLD staging and the severity of PH on echocardiography GOLD: Global Initiative for Chronic Obstructive Lung Disease; PH: pulmonary hypertension

PH Severity							
	GOLD Stage 1	GOLD Stage 2	GOLD Stage 3	GOLD Stage 4	Total	Chi-square test (χ2)	P-value
Normal	16 (88.9%)	27 (75.0%)	16 (48.5%)	1 (7.7%)	60 (60.0%)	36.195	<0.001
Mild	2 (11.1%)	9 (25.0%)	9 (27.3%)	6 (46.2%)	26 (26.0%)		
Moderate	0 (0.0%)	0 (0.0%)	6 (18.2%)	3 (23.1%)	9 (9.0%)		
Severe	0 (0.0%)	0 (0.0%)	2 (6.1%)	3 (23.1%)	5 (5.0%)		
Total	18 (100.0%)	36 (100.0%)	33 (100.0%)	13 (100.0%)	100 (100.0%)		

Comparison of the four subgroups of the GOLD staging in terms of systolic PAP

**Figure 3:**
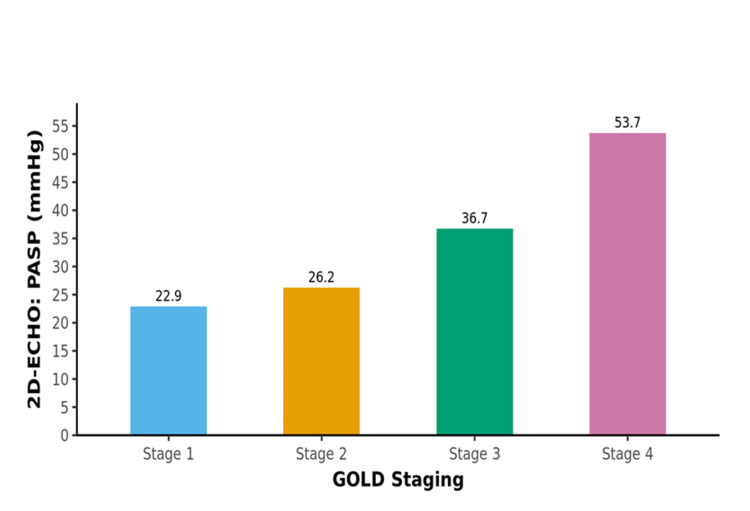
Association between GOLD staging and PASP GOLD: Global Initiative for Chronic Obstructive Lung Disease; PASP: pulmonary arterial systolic pressure

There was a significant difference between the four groups of GOLD staging in terms of systolic PAP (mm Hg) (χ2 = 26.415, p = <0.001), with the median systolic PAP (mmHg) being highest in the GOLD Stage 4 group. The bar graph below depicts the means of 2D echocardiography : systolic PAP (mm Hg) in the four different groups. (Table 8, Figure 3).

**Table 8:** Comparison of the four subgroups of the GOLD staging in terms of systolic PAP GOLD: Global Initiative for Chronic Obstructive Lung Disease; PAP: pulmonary arterial pressure; IQR: interquartile range

2D-ECHO: Systolic PAP (mmHg)						
	GOLD Stage 1	GOLD Stage 2	GOLD Stage 3	GOLD Stage 4	Kruskal-Wallis test (χ2)	p value
Mean (SD)	22.89 (6.85)	26.22 (9.64)	36.73 (18.74)	53.69 (18.96)	26.415	<0.001
Median (IQR)	20 (20-23.5)	20 (20-26.75)	35 (20-48)	46 (40-65)		
Range	15 - 45	20 - 50	18 - 78	25 – 85		

## Discussion

A total of 100 diagnosed patients with COPD were enrolled in the study. Out of these, very severe COPD was present in 13 patients (13%), severe COPD in 33 patients (33 %), moderate COPD in 36 patients (36%), and mild COPD in 18 patients (18%). According to the findings, 40% of COPD patients had PH, which is strikingly similar to the incidence of PH in earlier studies as shown below. Out of the 40 PH patients in our study, mild, moderate, and severe PH was seen in 26 patients (65%), nine patients (22.5%), and five patients (12.5%), respectively. The frequency of PH in moderate and severe COPD was 25% and 51.5%, respectively. As the COPD severity increased, prevalence and severity of PH were also found to be increased in the study and it was also observed that severe PH was present mostly in patients with severe COPD. Two patients (6.1%) had severe PH in moderate COPD, while three patients (23.1%) had severe PH in severe COPD. In a recent Indian study by Gupta et al. on 40 COPD patients, PH was found in 42.5% of cases with mild, moderate, and severe PH with prevalence rates of 25%, 10%, and 7.5%, respectively [11]. Our findings were comparable to this study. Gupta et al. concluded that prevalence and severity of PH increase with the severity of COPD. Similar conclusions were made by Scharf et al. and Doi M et al. where the severity of PH tends to correlate with the degree of airflow obstruction [12-13].

Previous studies have shown that in COPD and the frequency of severe PH in COPD ranges between 3-5% [14-15]. This finding was consistently found to be 5% in our study. In our study, it was found that the median duration of the history of COPD symptoms was higher in COPD with the PH group in comparison to the group with COPD without PH (p = 0.022) and this was statistically significant.

Also, it was noted in our study that 79% of the study participants were engaged in the agricultural sector. This was similar to a study by De Matteis [et al. in 2019 in which they found a higher COPD risk in subjects involved in the agricultural sector [16]. The higher percentage of study subjects with COPD from the agricultural sector might be explained by the location of study in rural central India with a predominantly agricultural economy.

Higham et al. concluded that in the majority of COPD patients, echocardiography can efficiently and reliably detect the presence and severity of pulmonary arterial hypertension, and given the negative effects of pulmonary arterial hypertension on morbidity and mortality, routine echocardiography in patients with severe COPD may be warranted [17].

In our study, it was noted that the mean systolic PAP estimated by echocardiography was progressively higher in higher GOLD stages of COPD (p = <0.001) and was found to be statistically significant, with the median systolic PAP (mmHg) being highest in the GOLD Stage 4 group. This was similar to the findings of the Rotterdam study in which they found that an absolute 10% decrease in FEV1 correlated with an estimated systolic PAP increase of 0.46 mm Hg [18].

As a result, in an ideal scenario, all COPD patients should be evaluated for PH using transthoracic Doppler echocardiography, which incorporates all of the necessary components for a screening and a diagnostic tool for early diagnosis, so that appropriate treatment may be recommended at the earliest. This will aid in the improvement of the quality of life and survival of these patients who battle a debilitating condition such as COPD.

Study limitations

This was a hospital-based study and only symptomatic patients with clinical, ECG, and echocardiographic findings of PH were included in the study. Right heart catheterization and measurement of pulmonary artery pressure, which is the gold standard to assess pulmonary hypertension, was not done due to hospital limitations. Initially, in our study, we had planned to do regular three and six-monthly follow-ups of the patients with repeat echocardiography to assess the disease progress, but we were unable to do so due to the coronavirus disease 2019 (COVID-19) pandemic situation that greatly affected both outpatient and inpatient numbers.

## Conclusions

Out of 100 COPD patients who fulfilled the inclusion and exclusion criteria in our study, the prevalence of PH was found to be 40% by Doppler echocardiography, out of which 26% had Mild PH, 9% had Moderate PH while 5% had Severe PH. As the COPD severity increased, the frequency and degree of PH were also found to be increased, and this difference was found to be statistically significant. Also, it was a notable finding in our study that patients with greater severity of airflow obstruction graded by GOLD staging were also found to have higher systolic PAP on echocardiography. Therefore, we conclude that echocardiography is an excellent tool for the detection of PH in COPD patients when special attention is given to the tricuspid regurgitation jet, right ventricular motility, and dilatation. It is our recommendation that all COPD patients with worsening dyspnea, should undergo echocardiography so as to detect PH, if present, at an early stage, since PH is a frequently observed finding in such patients.
